# Barriers and Facilitators of Implementing Automated Radiotherapy Planning: A Multisite Survey of Low- and Middle-Income Country Radiation Oncology Providers

**DOI:** 10.1200/GO.21.00431

**Published:** 2022-05-10

**Authors:** Gwendolyn J. McGinnis, Matthew S. Ning, Beth M. Beadle, Nanette Joubert, William Shaw, Christoph Trauernich, Hannah Simonds, Surbhi Grover, Carlos E. Cardenas, Laurence E. Court, Grace L. Smith

**Affiliations:** ^1^ Department of Radiation Oncology, The University of Texas MD Anderson Cancer Center, Houston, TX; ^2^ Department of Radiation Oncology, Stanford University, Palo Alto, CA; ^3^ Division of Medical Physics, University of Cape Town and Groote Schuur Hospital, Cape Town, South Africa; ^4^ Department of Medical Physics (G68), University of the Free State, Bloemfontein, South Africa; ^5^ Division of Medical Physics, Stellenbosch University, Tygerberg Academic Hospital, Cape Town, South Africa; ^6^ Division of Radiation Oncology, Stellenbosch University, Tygerberg Academic Hospital, Cape Town, South Africa; ^7^ School of Medicine, University of Botswana, Gaborone, Botswana; ^8^ Princess Marina Hospital, Gaborone, Botswana; ^9^ Department of Radiation Oncology, Perelman School of Medicine, University of Pennsylvania, Philadelphia, PA; ^10^ Botswana University of Pennsylvania Partnership, Gaborone, Botswana; ^11^ Department of Radiation Physics, The University of Alabama at Birmingham, Birmingham, AL; ^12^ Department of Radiation Physics, The University of Texas MD Anderson Cancer Center, Houston, TX; ^13^ Department of Health Services Research, The University of Texas MD Anderson Cancer Center, Houston, TX

## Abstract

Global access to radiotherapy (RT) is inequitable, with obstacles to implementing modern technologies in low- and middle- income countries (LMICs). The Radiation Planning Assistant (RPA) is a web-based automated RT planning software package intended to increase accessibility of high-quality RT planning. We surveyed LMIC RT providers to identify barriers and facilitators of future RPA deployment and uptake.

**METHODS:**

RT providers underwent a pilot RPA teaching session in sub-Saharan Africa (Botswana, South Africa, and Tanzania) and Central America (Guatemala). Thirty providers (30 of 33, 90.9% response rate) participated in a postsession survey.

**RESULTS:**

Respondents included physicians (n = 10, 33%), physicists (n = 9, 30%), dosimetrists (n = 8, 27%), residents/registrars (n = 1, 3.3%), radiation therapists (n = 1, 3.3%), and administrators (n = 1, 3.3%). Overall, 86.7% expressed interest in RPA; more respondents expected that RPA would be usable in 2 years (80%) compared with now (60%). Anticipated barriers were lack of reliable internet (80%), potential subscription fees (60%), and need for functionality in additional disease sites (48%). Expected facilitators included decreased workload (80%), decreased planning time (72%), and ability to treat more patients (64%). Forty-four percent anticipated that RPA would help transition from 2-dimensional to 3-dimensional techniques and 48% from 3-dimensional to intensity-modulated radiation treatment. Of a maximum acceptability/feasibility score of 60, physicians (45.6, standard deviation [SD] = 7.5) and dosimetrists (44.3, SD = 9.1) had lower scores than the mean for all respondents (48.3, SD = 7.7) although variation in scores by roles was not significantly different (*P* = .21).

**CONCLUSION:**

These data provide an early assessment and create an initial framework to identify stakeholder needs and establish priorities to address barriers and promote facilitators of RPA deployment and uptake across global sites, as well as to tailor to needs in LMICs.

## INTRODUCTION

Although radiotherapy (RT) is a mainstay of definitive and adjuvant cancer treatment, shortages of high-quality RT delivery exist globally.^1-4^ Global heterogeneity in use of modern, conformal RT techniques is attributed to inequities in national wealth and income, access to technology and clinical training, and availability of providers. An inadequate supply of medical physicists and dosimetrists, who possess the specific expertise required for modern conformal RT planning, including design of 3-dimensional (3-D) and intensity-modulated radiation treatment (IMRT) plans,^5^ further exacerbates these issues.

**CONTEXT**

**Key Objective**
Automated radiotherapy (RT) planning software has been developed to ease inequitable access to RT in low-resource environments. This multinational survey of experienced RT providers explores key facilitators and barriers to the implementation and uptake of automated RT planning software in oncology clinics in low- and middle-income countries.
**Knowledge Generated**
Key stakeholders to the use of automated RT planning software in low- and middle-income countries demonstrate a high level of interest in this technology for their clinics. However, important barriers to implementation remain.
**Relevance**
The use of automated RT planning could improve access to RT globally. Implementation across global sites will require tailoring to meet varying needs by provider role, practice site disease type burdens, and infrastructural resources.

The development of automated RT planning tools is a potential initiative to address the global shortage of RT planning technology and professional expertise. The Radiation Planning Assistant (RPA) has been developed to improve the availability of high-quality radiation in low-resource settings, and it has been previously described.^4^ This system is web-based and fully automated (including built-in quality assurance) for steps of RT planning that traditionally require in-person physicist expertise, along with physician and dosimetrist roles: isocenter marking, target contouring, beam design, and RT plan optimization. Optimizing radiation dose distribution and reducing heterogeneity are hallmarks of high-quality 3-D and IMRT plan design. RPA has already demonstrated technical effectiveness through robust optimization and feasibility for generating cervical, head and neck, and breast cancer RT plans.^2-4^

In low-resource settings, RPA's automation and web-based computing have the potential to mitigate staff and educational/training shortages. In doing so, treating facilities using RPA further have the potential to increase patient throughput (volume) and systematic planning processes (quality). However, the integration of the RPA may also face challenges, including concerns for job stability, internet availability, trust of an automated system, and appropriate disease paradigms, among others. To assess these potential benefits and challenges, we surveyed radiation oncology providers in multiple centers in sub-Saharan Africa and Central America.

## METHODS

### Study and Survey Design

This study was approved by the University of Texas M.D. Anderson Cancer Center institutional review board. A total of 33 RT providers at facilities in four countries of sub-Saharan Africa (Botswana, South Africa, and Tanzania) and Central America (Guatemala) who were undergoing an interactive learning and simulation session for use of RPA were invited to participate in the postsession survey, with N = 30 consented to participate after reviewing a brief study description and questionnaire statement. The teaching and simulation session consisted of an approximately 60-minute educational session on principles of automated remote planning and included a simulation of the web-based planning process for head and neck and breast IMRT plans, including isocenter marking, target contouring, beam design, and RT plan optimization steps. Initial sessions and paper survey in Botswana were conducted in person on-site. Because of COVID-19 pandemic travel restrictions, subsequent sessions in South Africa, Tanzania, and Guatemala were conducted over Zoom, with the instructor (L.E.C.) in the United States and the survey provided as an electronic form. Respondents completed the anonymous survey immediately upon ending the in-person or remote learning and simulation session.

The English language survey was initially piloted in five respondents, along with qualitative open-ended and cognitive interview questions to ensure the relevance of survey questions to respondents' practice environment; comprehensiveness of content; and optimization of comprehensibility, acceptability, scoring system, recall period, and survey burden. After this pilot period, questions on practice characteristics (eg, patient volume and time needed for RT planning) were excluded because of survey burden expressed by pilot respondents. Questions on provider characteristics, attitudes, expectations about barriers and facilitators of deployment and utilization, and training and support needs were retained. Questions were added on (1) RPA user experience (ie, ease of use of web page navigation), specific tasks (computed tomography upload, service request, contour download, and plan download), and RPA registration and (2) feasibility and acceptability of RPA implementation. Providers were asked to rate the RPA on a scale of 1-5 (with 1 being completely disagree and 5 being completely agree) on 12 measures (total score 60) using the validated Acceptability of Intervention Measure, Intervention Appropriateness Measure, and Feasibility of Intervention Measure.^6^ This measure has previously demonstrated correlation with success of implementation.^7^

### Statistical Analysis

Analyses were conducted on the basis of the denominator of providers who answered each question since some questions were developed after the initial pilot session and therefore not answered by the initial five participants. Descriptive statistics summarized demographic characteristics and responses. Acceptability scores by provider role were compared using the one-way analysis of variance test. Facilitators and barriers to RPA uptake by provider role were analyzed by Fisher's exact test. A two-sided 5% level of significance was used. All statistical analyses were performed using SPSS Version 24 (IBM Corp, Armonk, NY).

## RESULTS

### Provider Characteristics

Detailed provider characteristics are shown in Table 1. The majority of respondents were age between 31 and 50 years (73.3%) and had been in practice in their current role for more than 5 years (72.4%). Most respondents were currently practicing in either South Africa (43.3%) or Guatemala (36.7%). Fewer participants were practicing in Botswana (16.7%) and Tanzania (3.3%). The most frequently represented providers were radiation oncologists (33.3%), medical physicists (30.0%), and medical dosimetrists (26.7%).

**TABLE 1:**
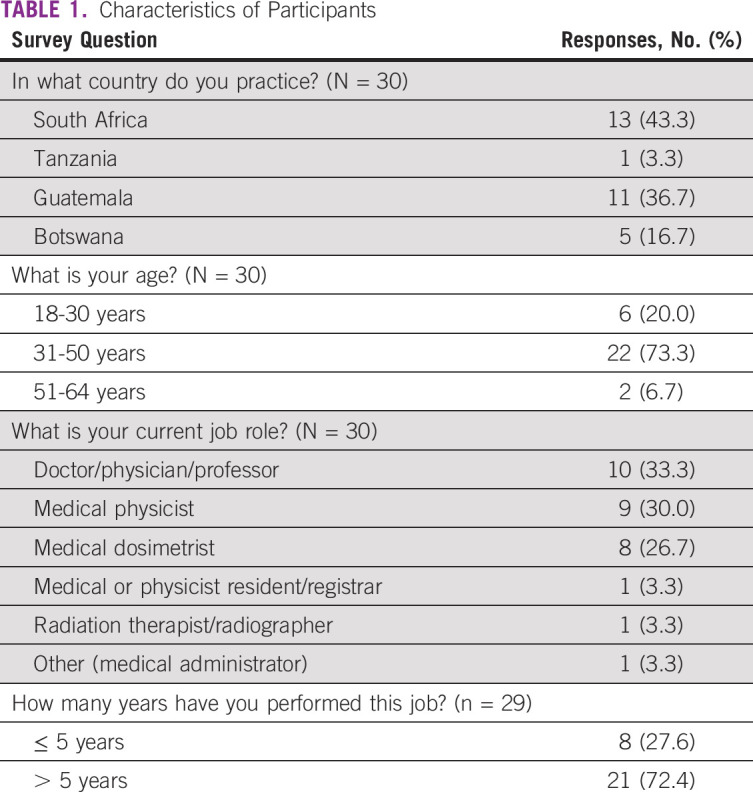
Characteristics of Participants

### Interest in the RPA

**FIG 1:**
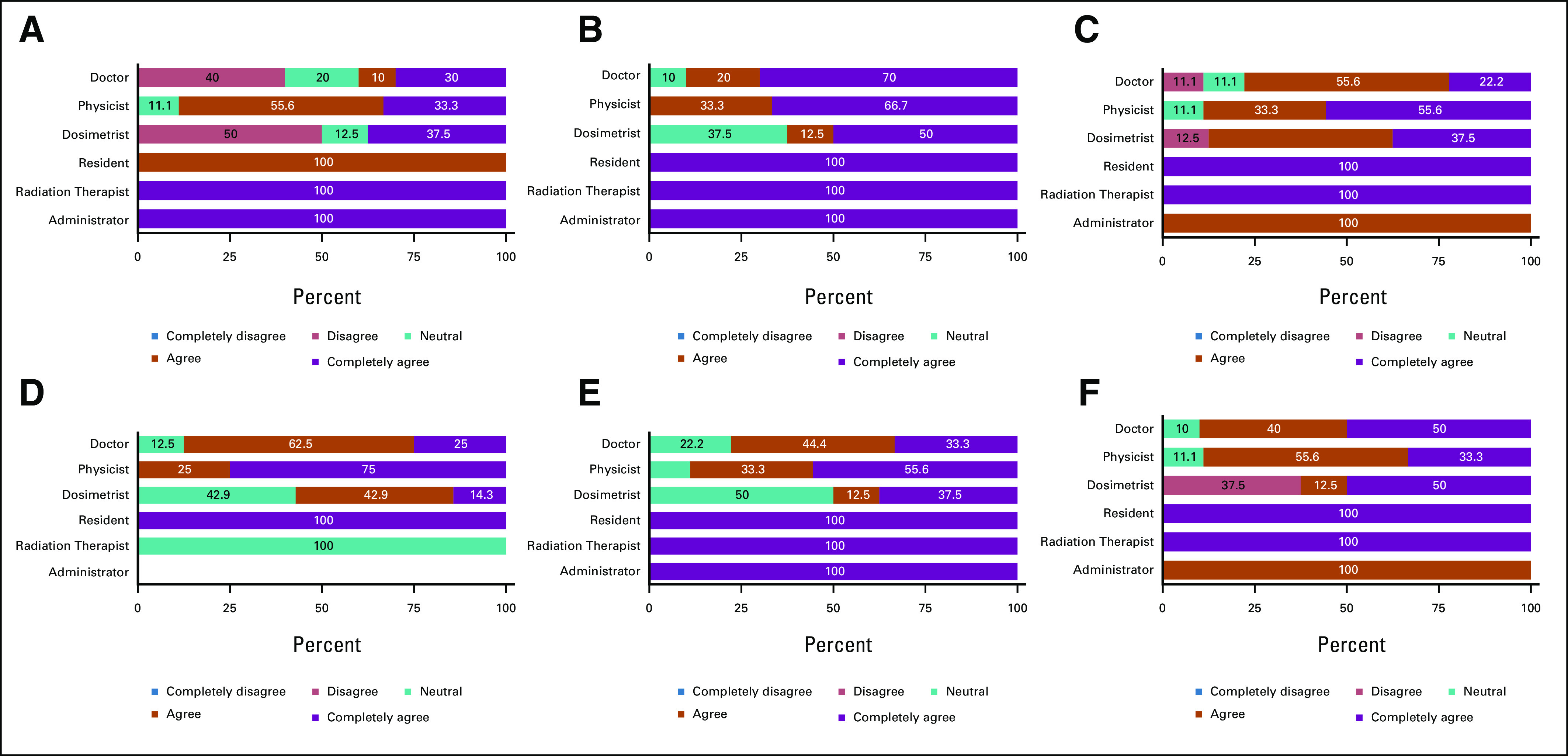
Provider attitudes on RPA. Providers were asked to respond to statements regarding their opinions about the RPA on a scale of 1-5, with 1 being completely disagree and 5 being completely agree. Data are shown as percent per value chosen separated by provider role (physician n = 10, physicist n = 9, dosimetrist n = 8, resident n = 1, RTT n = 1, and administrator n = 1). Results from Fisher's exact test show (A) *P* = .945 (I think it is possible to begin using the RPA in my practice now); (B) *P* = .060 (I have a high interest level in the RPA); One participant (role = administrator) did not respond to the prompt, so this bar is not shown; (C) *P* = .703 (I think it would be easy for everyone in my clinic to use the RPA); (D) *P* = .845 (I think it is possible to begin using the RPA in my practice within 2 years); (E) *P* = .310 (I would like to use the RPA to plan radiotherapy for the cancer patients I treat); and (F) *P* = .711 (I think it would improve my workflow to use the RPA). RPA, Radiation Planning Assistant.

There were no significant differences by provider role, age, or time in practice on any of the interest-related statements regarding the RPA (Fig 1). Of respondents, 60.0% either agreed or completely agreed that the RPA could be used immediately in their practice, whereas 13.3% felt neutral and 26.7% disagreed (*P* = .10). When asked if providers thought that the RPA could be used within 2 years in their practice, 80.0% agreed or completely agreed, 20.0% remained neutral, and none disagreed (*P* = .60). Most providers either agreed or completely agreed (75.9%) that they would like to use the RPA to plan RT for the patients with cancer they treat and most agreed or completely agreed (86.7%) with the statement that they had a high level of interest in the RPA (*P* = .70). Many providers agreed or completely agreed with the statement that the RPA would improve their workflow (83.4%; *P* = .31). Eighty-six percent either agreed or completely agreed that the RPA would be easy for everyone in their clinic to use (*P* = .85).

### Anticipated Barriers to and Facilitators of Implementation

Details on barriers and facilitators by provider role are given in Tables 2 and 3. Providers frequently selected anticipated facilitators of implementation related to efficiency of planning (decreasing workload [83.3%], decreasing time to plan [76.7%], and decreasing time it takes for patients to begin treatment [63.3%]) and clinical throughput (allowing more patients to be treated per year [60.0%]). Responses were mixed regarding benefits to consistency (of treatment plants between patients with cancer [66.7%], of contouring of targets between providers [53.3%], of contouring normal tissues between providers [50.0%], and of treatment plans between providers [43.3%]). Providers were less likely to anticipate modern treatment enhancement (decreased cost [13.3%], facilitating change from 2D to 3D treatments [36.7%], and facilitating change from 3D to IMRT treatments [40.0%]).

**TABLE 2:**
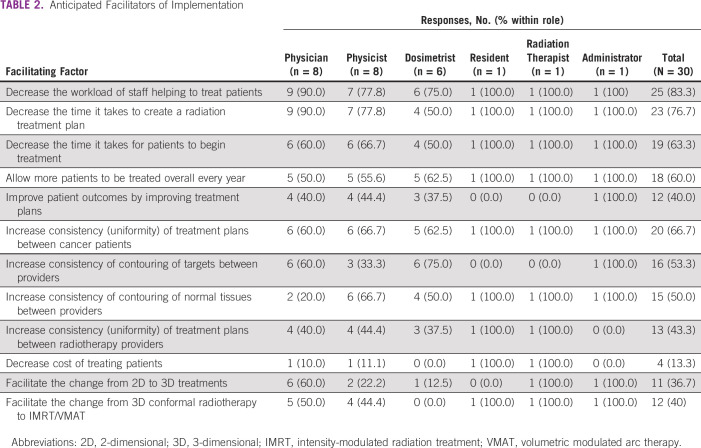
Anticipated Facilitators of Implementation

**TABLE 3:**
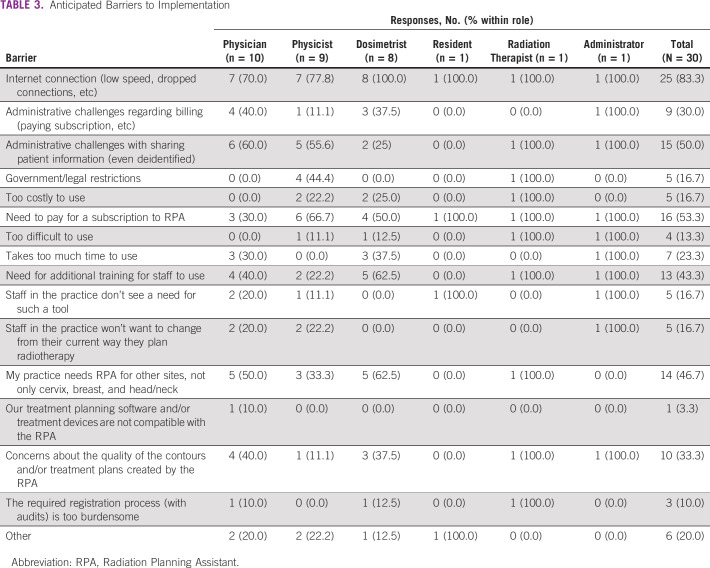
Anticipated Barriers to Implementation

Providers frequently anticipated that internet connection would be a barrier (83.3%). Responses were mixed on administrative challenges (regarding billing [30.0%] or patient information [50.0%]), the need to pay for a subscription (53.3%), additional training needs (43.3%), and need for additional treatment sites (46.7%). Providers were less likely to see initial cost (16.7%), government restrictions (16.7%), difficulty of use (13.3%), or quality of results (33.3%) as barriers to RPA uptake. Specific concerns offered in freeform responses included importation into a closed system, native language, incompatibility with equipment, administrative red tape, buy-in from staff, and multiple responses listing the concern for missed training opportunities for learners at academic centers.

### Needs for Training and Ongoing Support

**FIG 2:**
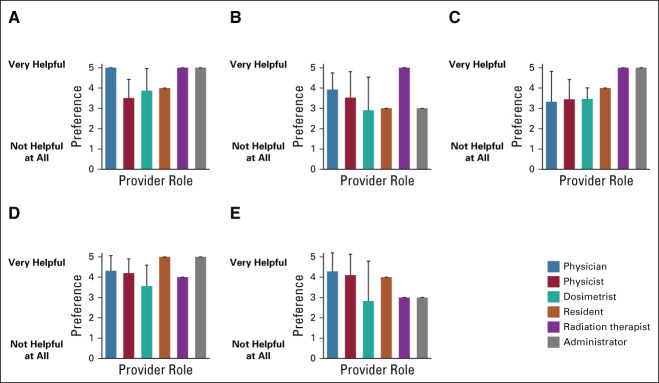
Provider preferences on training materials. Providers were asked if certain types of training materials would be useful to be trained on the RPA on a scale of 1-5, with 1 being not helpful at all and 5 being very helpful. Mean scores and standard deviations are shown by provider role. Preference for (A) in-person trainers present on-site at your institution did differ significantly by role (*P* = .006; Fisher's exact test). There were no significant differences in training type preference by role for (B) printed materials (books, articles, pamphlets, etc; *P* = .918), (C) remote trainers (*P* = .465), (D) online training (*P* = .205), or (E) video tutorials (*P* = .132). RPA, Radiation Planning Assistant.

**FIG 3:**
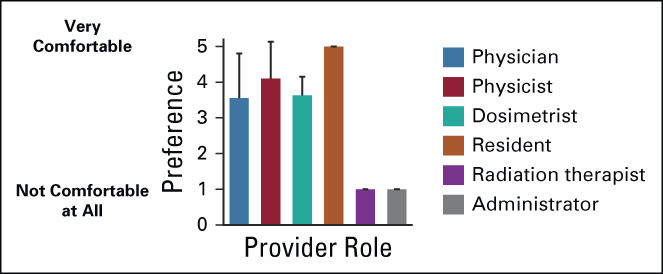
Provider comfort after online training program. Providers were asked to rate their comfort level using the RPA after completing only an online training program. Data are shown as the mean chosen value with standard deviation. The comfort level was significantly different by role (*P* = .035). RPA, Radiation Planning Assistant.

Most participants reported that they would find printed materials (86.7%), on-site in-person training (93.3%), off-site in-person training (86.7%), interactive online training (90.0%), and online video tutorials (96.7%) helpful as resources for initial training. Preference for initial training is shown by role in Figure 2. For ratings of helpfulness (1, not helpful at all to 5, very helpful), on-site in-person training (mean 4.3, standard deviation [SD] = 1.0) scored as the most helpful resource with rating varying significantly by provider role (*P* < .01). This was followed by interactive online training (mean 4.2, SD = 0.8), online video tutorials (mean 3.9, SD = 1.3), off-site in-person training (mean 3.7, SD = 1.1), and printed materials (mean 3.7, SD = 1.2), all of which did not show significant differences in preference by role. Forty-eight percent of providers reported that they had not previously used online training to learn about software for treatment planning. When asked how comfortable they would be using the RPA after completing only an online training program on a scale of 1-5 (5 being very comfortable), providers rated a mean score of 3.8 (SD = 1.3). However, this varied significantly by role (Fig 3; *P* < .05). Freeform responses for other initial training types requested included flowchart, guidelines, webinar, operating procedure, frequently asked questions database, and practice runs.

**FIG 4:**
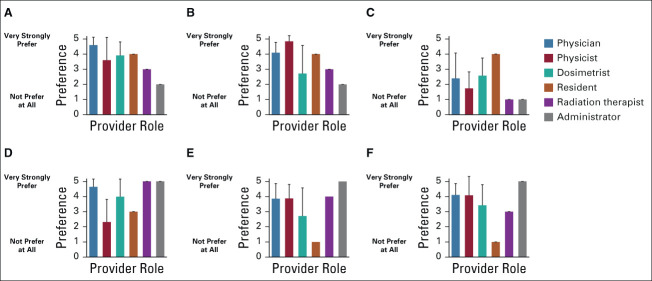
Provider preferences on ongoing support materials and resources Providers were asked which types of ongoing support they would prefer for the RPA on a scale of 1-5, with 1 being not prefer at all and 5 being very strongly prefer. Mean scores and standard deviations are shown by provider role. Preferences for (A) online chat (*P* = .045) and (B) e-mail (*P* = .023) support did differ significantly by role (Fisher's exact test). There were no significant differences in opinion by role for (C) telephone (*P* = .546), (D) in-person (*P* = .486), (E) scheduled online (*P* = .466), or (F) online discussion groups (*P* = .119). RPA, Radiation Planning Assistant.

Most participants reported that they would find online chat help (93.3%), e-mail help (80.0%), scheduled (eg, weekly) online classroom/support group (80.0%), in-person technical or educational support (80.0%), a telephone helpline (76.7%), and online discussion groups (eg, google groups; 73.3%) helpful as resources for ongoing support when using RPA. Preference for ongoing support is shown by role in Figure 4. In-person technical support (mean 4.0, SD = 1.4) was rated as the most preferred on a scale of 1-5 (with 5 being very strongly preferred) followed by online chat (mean 3.9, SD = 1.2), e-mail (mean 3.9, SD = 1.3), online discussion (mean 3.9, SD = 1.3), scheduled online (mean 3.7, SD = 1.5), and telephone (mean 2.7, SD = 1.4) support. There were significant differences by role in preference for online chat (*P* < .05) and e-mail (*P* < .05), but not for telephone (*P* = .546), in-person (*P* = .486), scheduled online (*P* = .466), or online discussion groups (*P* = .119). Freeform suggestions for other types of ongoing support included on-site visit to the designing institution, a reference manual, remote courses, and WhatsApp (a free, cross-platform centralized instant messaging and voice-over-internet protocol service).

### Acceptability, Feasibility, and Appropriateness of RPA Implementation

**FIG 5:**
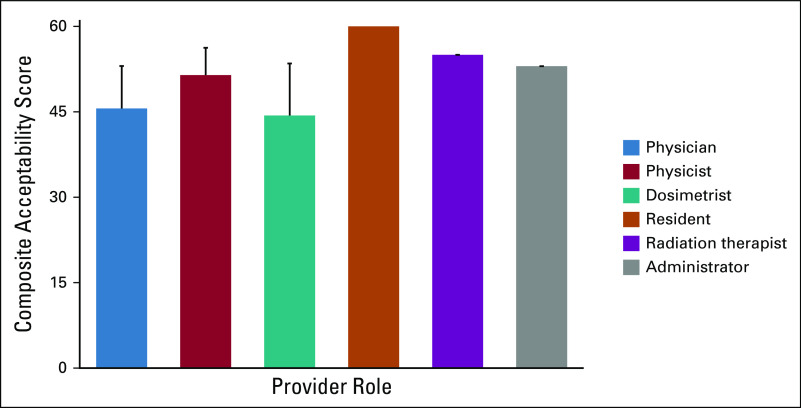
Overall median acceptability, feasibility, and appropriateness score by provider role. Twenty-five providers from three countries were asked to rate RPA acceptability, feasibility, and appropriateness on a scale of 1-5 on 12 measures (total score 60) using the validated AIM, IAM, and FIM.^6^ This validated measure capturing the three dimensions of acceptability, feasibility, and appropriateness has been correlated with success of implementation.^7^ There were no significant differences by provider role (*P* = .213). AIM, Acceptability of Intervention Measure; FIM, Feasibility of Intervention Measure; IAM, Intervention Appropriateness Measure; RPA, Radiation Planning Assistant.

Mean provider summary scores across 12 domains of acceptability of the RPA by role are shown in Figure 5. Of a maximum total score of 60, the mean acceptability score across all surveyed providers was 48.3 (SD = 7.7). Although mean scores for physicians (45.6, SD = 7.5) and dosimetrists (44.3, SD = 9.1) were the lowest for any provider type, these differences did not achieve statistical significance (*P* = .213).

### Additional Qualitative Themes About RPA Implementation

Key quotes are provided in Table 4. Themes that emerged included enthusiasm for an automatic planning system to increase the capacity for patient treatment volume tempered by fears about the potential financial burden of new technology implementation and novel themes raised about the risk of replacing in-person learning experiences and training opportunities for treatment planning and the threat of future decreased need for staff.

**TABLE 4:**
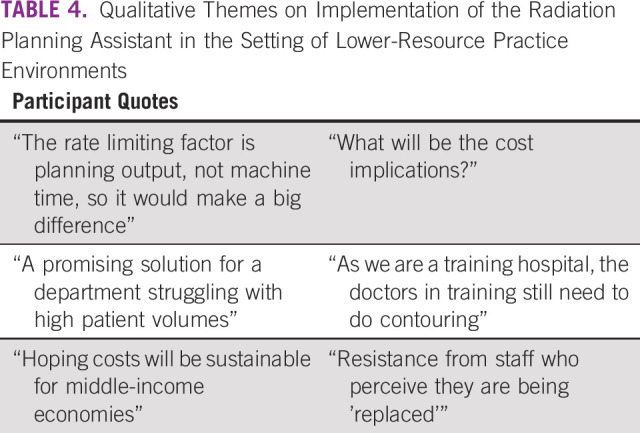
Qualitative Themes on Implementation of the Radiation Planning Assistant in the Setting of Lower-Resource Practice Environments

## DISCUSSION

Our study provides initial novel insights into anticipated barriers, facilitators, and needs in considering adoption of the RPA, on the basis of respondents representing experienced radiation therapy providers across multiple low- and middle-income country (LMIC) international sites. Among respondents, there were many positive impressions of the RPA, with high interest and expectations about the impact it might have on their clinical practice and on their patients. However, there remain significant areas of concern regarding implementation including logistical barriers, especially internet connectivity and cost, and a need for additional training and ongoing support, as well as concern over the impact of automated treatment planning on the training of future generations of radiation therapy providers.

Future considerations for implementation. Originally, the RPA was designed as a stand-alone system that require setup in each clinic. This approach was abandoned early on, as it was determined that this approach would add significant additional cost for both installation and service. Instead, the RPA is now designed as a web-based tool, as this approach may help reach as many clinics as possible, while minimizing cost. Nevertheless, this strategy introduces internet connectivity as a potential hurdle, and this barrier was selected as a concern by 80% of respondents. Solutions to enhance the robustness of the system to poor internet bandwidth will be needed to facilitate RPA implementation, including the possibility of alternative solutions such as satellite-based internet to support the system.

Cost for using and maintaining RPA was identified by 60% of the respondents as being a potential hurdle to use. Although the artificial intelligence–based RPA solution for treatment planning has sought to minimize expensive staffing resources and costs, still, this frequently expressed concern by survey respondents highlights the need to keep this service as low cost as possible for successful LMIC uptake. In addition, the specific sources of costs will need to be delineated, as these may differ by setting on the basis of existing infrastructure, government, and regulatory, legal, and health care systems. In addition, cost concerns highlighted by RPA uptake may not be entirely separable from the overall cost concerns for uptake and expansion of new radiation treatment technologies to advance modern planning and delivery approaches.

In conclusion, these data provide an early assessment and create an initial framework to identify stakeholder needs and establish priorities to address barriers and promote facilitators of RPA deployment and uptake in LMICs. Results suggest that implementation across global sites will require tailoring to meet varying needs by provider role, practice site disease type burdens, and infrastructural resources.

## APPENDIX. Collaborators

Dawn Balang, Riette Burger, Memory Bvochora-Nsingo, Sebathu Chiyapo, L'lani Cooper, Sameera Dalvie, Sonia Davila, Vicky de Falla, Nazia Fakie, Monique du Toit, Osmar Hernandez, Magda Heunis, Otto Hurtarte, Milton Ixquiac, Suzaan Lategan, Lorens Strauss, Komeela Naidoo, Remigio Makufa, Thokozani Mkhize, Kirk Najera, Hildah Napo, Sarah K Nyagabona, Odirile Obuseng, Jeannette Parkes, Melanie Pelser, Anayansi Ramirez, Ricus van Reenen, Edgar Ruiz, Carlos Salazar, Alicia Sherriff, Lilliana Sobrevilla, Brenda van Urk, Angel Velarde, Julie Wetter.
